# Isolation and Genomic Characterization of an *Acinetobacter johnsonii* Bacteriophage AJO2 From Bulking Activated Sludge

**DOI:** 10.3389/fmicb.2019.00266

**Published:** 2019-02-20

**Authors:** Niansi Fan, Min Yang, Rencun Jin, Rong Qi

**Affiliations:** ^1^College of Life and Environmental Sciences, Hangzhou Normal University, Hangzhou, China; ^2^State Key Laboratory of Environmental Aquatic Chemistry, Research Center for Eco-Environmental Sciences, University of Chinese Academy of Sciences, Beijing, China

**Keywords:** *Acinetobacter johnsonii*, bacteriophage, phage AJO2, activated sludge, genome sequence

## Abstract

A novel *Podoviridae* lytic phage AJO2, specifically infecting *Acinetobacter johnsonii*, was isolated from bulking activated sludge. The one-step growth experiment showed that the latent period and burst size of AJO2 were estimated to be 30 min and 78.1 phages per infected cell, respectively. The viability test indicated that neutral conditions (pH 6–8) were table for AJO2 survival, while it was sensitive to high temperature (≥60°C) and ultraviolet treatment (254 nm). Genomic sequencing revealed that the AJO2 had a linearly permuted, double-stranded (ds) DNA consisting of 38,124 bp, with the G+C content of 41 mol%. A total of 58 putative open reading frames (ORFs), 11 pairs of repeats and 11 promoters were identified. The AJO2 genome had a modular gene structure which shared some similarities to those of *A. baumanii* phages. However, genomic comparative analysis revealed many differences among them, and novel genes were identified in the AJO2 genome. These results contribute to subsequent researches on the interaction between bacteriophages and hosts in wastewater treatment, especially during the bulking period. Additionally, the newly isolated phage could be a good candidate as a therapeutic agent to control nosocomial infections caused by *A. johnsonii.*

## Introduction

As a common problem in activated sludge systems, sludge bulking draws extensive attention worldwide because of its serious consequences, such as poor sludge settle ability, water quality deterioration, sludge loss, or even system failure (Wagner, 1982; Madoni et al., 2000; Martins et al., 2004). Most sludge bulking events are caused by the overgrowth of filamentous bacteria, such as *Microthrix parvicella*, *Nostocoida limicola*, and *Nocardia nova* (Jenkins et al., 2004; Wang et al., 2014). In addition, non-filamentous bacteria also play a vital role in system function maintenance, such as *Nitrospira*, *Nitrosomonas*, polyphosphate-accumulating organisms (PAOs) and glycogen-accumulating organisms (GAOs) (Levantesi et al., 2010; Nielsen et al., 2012). The dynamic equilibrium of the microbial community is an important prerequisite for the operational stability of wastewater treatment plants (WWTPs).

Bacteriophages are the most abundant biological entities in the environment (Chibani-Chennoufi et al., 2004; Mann, 2005). Phages appear to be active components of activated sludge systems and phage-mediated bacterial mortality has the potential to influence treatment performance by influencing the bacterial abundance (Withey et al., 2005). On the one hand, analyzing the dynamic changes of bacteriophages that infect key functional groups contributes to understanding the malfunctions in WWTPs. On the other hand, the host specificity of phages makes them a potentially efficient tool to eliminate bulking-related filamentous bacteria. For example, the HHY-phage lysing *Haliscomenobacter hydrossis* (Kotay et al., 2011), SNP-phage lysing *Sphaerotilus natans* (Kotay et al., 2010) and THE1 lysing *Tetrasphaera jenkinsii* (Petrovski et al., 2012) have been isolated from activated sludge with the goal of filamentous bulking control. However, the physiological and genomic characterization of phages is incomplete and phage-host interaction mechanisms are still unclear. The main reason is that only a few phages have been isolated and sequenced, limiting its characterization and further application of “phage therapy.” Therefore, it is urgent to isolate and genetically characterize more phages in activated sludge, especially during the bulking period.

In this study, phage AJO2 specific to *A. johnsonii* was isolated from bulking activated sludge. Its biological characteristics and genome sequence were also determined. Comparative analyses of AJO2, the previously-isolated phage AJO1 (Fan et al., 2017) and other phages with closely phylogenetic relationships were conducted on the basis of their morphologies, host ranges, burst sizes and genomic features. Results showed that AJO1 and AJO2 shared some similarity, but their DNA sequences and annotations revealed many differences and novel attributes. The new phage provides a valuable resource for further investigations on its physiochemical properties and relationships between phages and hosts.

## Materials and Methods

### Bacterial Strains and Culture Conditions

A total of 44 strains were isolated from a large municipal WWTP in Beijing during a bulking period (SVI = 180 mL g^-1^). 16s rRNA gene amplification and sequencing were performed for their precise identification. As one of the dominant cultivatable microorganisms, *A. johnsonii* strain Pt405 was selected as the host bacterium for phage isolation, enrichment and characterization. This strain could form 0.5 to 2.0 mm in diameter, milk-white, opaque, round colonies on LB agar at 30°C. Another 17 strains were used for host range determination in the study (Supplementary Table S1). Nine strains from different phyla, such as *Kocuria rosea*, *Bacillus thuringiensis*, *Flavobacterium*, *Delftia tsuruhatensis* strain M6 etc., were also isolated from the bulking sludge in the same period. Eight strains within *Acinetobacter* were obtained from China General Microbiological Culture Collection Center (CGMCC), including *A. tandoii*, *A. lwoffii*, *A. junii*, *A. haemolyticus*, *A. calcoaceticus*, *A. baumannii*, *A. radioresistens*, and *A. bouvetii*. These strains were cultured on nutrient agar at 30°C. The turbidimetric method was adopted to monitor bacterial growth through measuring optical density at 600 nm (OD_600_). All strains were stored at -20°C for the short term and at -80°C with the equivalent 30% glycerol for the long term.

### Phage Isolation and Transmission Electron Microscopy (TEM)

The isolated *A. johnsonii* strain Pt405 was used for the isolation of a lytic bacteriophage. A volume of 20 L fresh sludge sample was centrifuged at 8000 × *g* for 20 min and filtered in turn through 0.45 and 0.22 μm pore size syringe filter (Millipore, United States) to remove bacterial debris. For further enrichment, tangential flow ultrafiltration (Vivaflow 200, Sartorius, Germany) was performed and the final volume of filtrate was 150 mL. Fresh host strain was propagated in LB broth for 4--6 h at 30°C. Subsequently, 50 mL bacterial suspension and 1 mL filtrate were gently mixed, and allow to stand for at least 1 h at room temperature with the aim of better absorption between phage and host. Afterward, the mixture was cultured by shaking (100 rpm) at 30°C for 6 h. Modified double-layer agar (DLA) assays (Adams, 1959) were performed to confirm the plaques. In brief, equal volumes of bacterial suspension and enriched filtrate were added into warm LB agar (containing 0.7% agar), and poured on a prepared LB agar plate (containing 1.5% agar). Bacteriophage plaques could be observed after incubating at 30°C overnight, and single plaque purification was performed six times according to Fan et al. (2017). The purified phages were stored in SM buffer (10 mM Tris-HCl, pH 7.5, 10 mM MgSO_4_⋅7H_2_O and 100 mM NaCl) at 4°C for short periods.

Phage particles were precipitated according to the NaCl/PEG protocol (Petrovski et al., 2012), and observed using transmission election microscopy (H7500, Hitachi, Japan) at 100 kV as described by Fan et al. (2017). The morphological features of phages were observed, and the family to which they belong could be determined.

### General Characteristics of Phage

The one-step growth curve was determined at the multiplicity of infection (MOI) of 0.001 according to a previous description (Fan et al., 2017). Spectrophotometry (Jing Dan et al., 2013) and the spot-test method (Chopin et al., 1976) were performed to determine the host range. Briefly, bacterial suspensions (OD_600_∼0.2) were mixed with 100 μL phage stock, and OD_600_ of each mixture was measured using spectrophotometer after 40 min incubation at 30°C. In the spot-test assay, 50 μL phage stock (>10^7^ PFU mL^-1^) was spotted on prepared agar plates spread with the tested stains.

### Resistance to Various Environmental Factors

Considering the application prospects, the phage susceptibility to environmental changes was evaluated, including temperature, pH and ultraviolet (UV) light. In the thermal treatment experiment, 1.5 mL phage stock was incubated at 55, 60, and 70°C water bath for 5, 10, 15, and 20 min, respectively. In the pH test, phage stocks were incubated in media with pH of 2.0–11.0 (1.0 interval) at 30°C. Advanced oxidation processes like UV photolysis have been widely used in wastewater treatment (De la Cruz et al., 2013; Deng and Zhao, 2015), thus UV light was selected as one of the environmental factors in this study. In the UV inactivation experiments, phage stocks were exposed under a vertical UV light (254 nm, 15W) for 10, 30, 60, 120, and 180 s, respectively. Phage titers of each set were determined using the double-layer method.

### Genomic Sequencing and Annotation

Phage DNA was extracted using SDS/proteinase K as previously described by Petrovski et al. (2011). DNase I (Sigma), RNase A (Sigma) and Mung Bean Nuclease (Takara) were used to determine the nucleic acid type with the working concentration of 1 unit μL^-1^ at 37°C for 1 h. Mung Bean Nuclease is a single-strand-specific nuclease. Enzyme *Spe*I (37°C for 3 h) was also used to determine whether the genome was linear or circular.

Genomic DNA was sequenced at Novogene Company (Beijing, China). Reads were assembled using SOAP *de novo* software. Open reading frames (ORFs) were predicted using ORF Finder ^1^ and the GeneMarkS interface^2^. Sequence similarity was analyzed using the BLAST X interface against a non-redundant database. Putative functions were predicted based on the BLAST P algorithm (Parsley et al., 2010; Jebri et al., 2016). The conserved motifs were identified using the conserved domain database (CDD)^3^, and the Pfam database^4^ was used for predicted protein family allocation (Finn et al., 2010). The putative rRNA, tRNA, transfer mRNA (tmRNA) and sRNA were screened using RNAmmer (Lagesen et al., 2007), rRNAscan-SE (Lowe and Eddy, 1997; Schattner et al., 2005) and Rfam (Burge et al., 2012). Putative promoters and terminators were predicted by neural network promoter prediction and the FindTerm web tool^5^ with an energy threshold value of -15. In addition, putative restriction enzyme cutting sites were predicted by NEBcutter V2.0 program^6^ (Vincze et al., 2003). Phylogenetic analysis was performed using MEGA7 via the neighbor-joining method (Kumar et al., 2016). Based on the annotated phage genome, GenBank files were created with Sequin ^7^, and the sequences of several fragments from phage genome were deposited in the GenBank database.

## Results and Discussion

### Isolation and General Features of Bacteriophage AJO2

**FIGURE 1:**
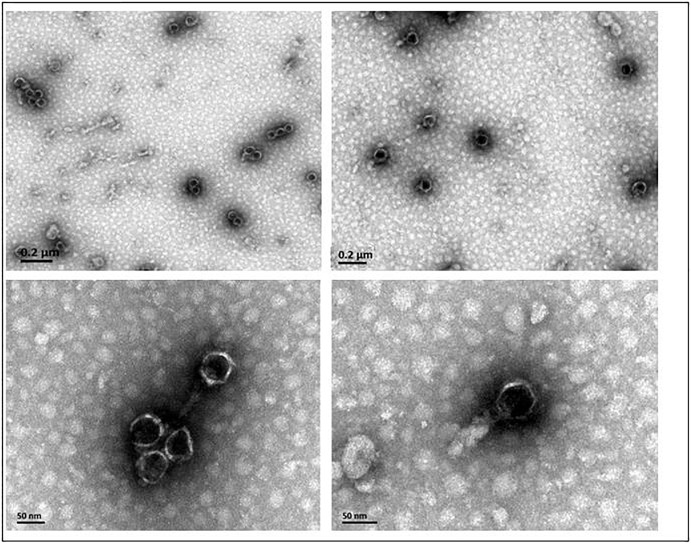
The morphology of phage AJO2 observed by TEM.

Phage AJO2 was isolated from a bulking sludge sample as a clear-plaque former against *A. johnsonii* strain Pt405. TEM observations (Figure 1) revealed that AJO2 had a collar and tail structure, appearing to belong to the order Caudovirales (Ackermann, 1998). Its icosahedral head (50 ± 2 nm) and short tail (6 ± 2 nm) is a typical morphology of the family *Podoviridae*. Compared with other *Podoviridae* phages, AJO2 was relatively smaller than Acibel007 (60 nm head and 10 nm tail) (Merabishvili et al., 2014), ϕAB6 (60 nm head and 11 nm tail) (Lai et al., 2016) and Abp1 (55 nm head and 15 nm tail) (Huang et al., 2013).

The one-step growth curve (Supplementary Figure S1) indicated that the latent period of AJO2 was around 30 min, and the rise period was completed at 80 min. The burst size was calculated to be 78.1 phages per infected cell. Both spot assay (Supplementary Table S2) and spectrophotometry results (Supplementary Figure S2).

### Viability of AJO2 Under Various Conditions

**FIGURE 2:**
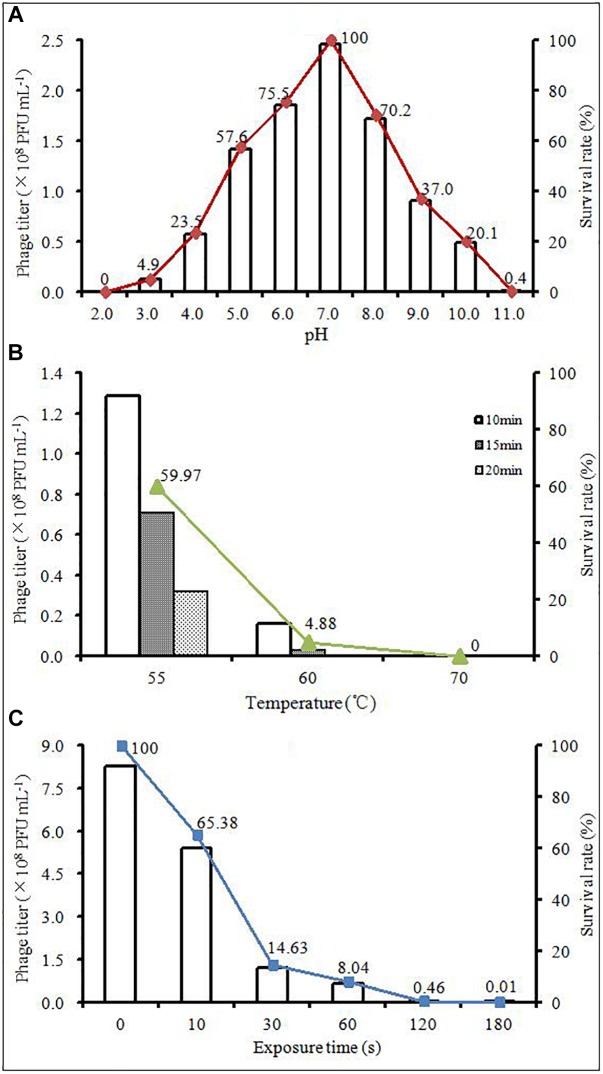
Changes in the titer of AJO2 under different **(A)** temperature, **(B)** pH, and **(C)** time of exposure to UV light at 254 nm.

The effects of pH, thermal and UV light on phage AJO2 were evaluated in this study. In the pH tests (Figure 2A), the survival rate of AJO2 maintained a level above 70% over a pH range of 6.0–8.0 and the optimal pH was 7.0. Compared with alkaline conditions (pH > 8.0), the activity of AJO2 was less affected under weakly acidic conditions (5.0–6.0). Pre-experiments showed that AJO2 was still infective after incubation at 46°C for 30 min, so higher temperatures were chosen in the thermal tests (Figure 2B). AJO2 could maintain a relatively high level of titer at 55°C, but its survival rate dramatically decreased below 5% at 60°C for 15 min. When the temperature increased to 70°C, AJO2 almost lost viability, indicating that the survival rate of AJO2 decreased with increasing temperature and exposure time. Similarly, AJO2 was also sensitive to UV light of 254 nm (Figure 2C). The survival rate of AJO2 quickly decreased from 100 to 14.63% within 30 s, and only 0.01% of phages remained infective after 3 min treatment. Although, this is a rough evaluation on the effect of UV light, it could reflect the AJO2 sensitivity to UV light to a certain extent, which provides valuable information for its practical application in the future. Therefore, neutral conditions were suitable for AJO2 survival, and it could tolerate relatively high temperatures (<60°C) and a short-term exposure to the UV light.

### Genome Analysis and Annotation

**FIGURE 3:**
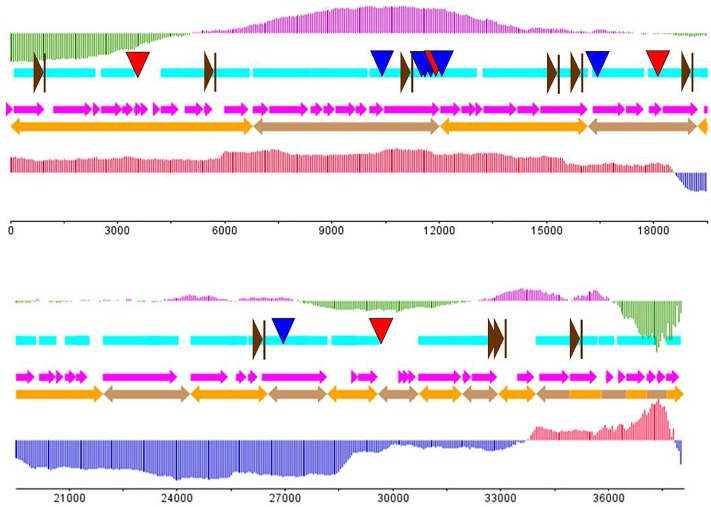
The putative genome draft of phage AJO2. Sky-blue band indicates gene-encoding regions in the AJO1 genome; orange arrows indicate assembled scaffolds and pink arrows represent predicted ORFs in the AJO1 genome. The GC skew is calculated as (G-C)/(G+C) and the GC plot shows GC% content. The upper vertical line with fixed intervals represents the GC skew (purple for positive and green for negative); the lower vertical lines represent the GC plot (red for above-average and blue for below-average). The downward arrows give the locations of repeats (blue for LINE and red for SINE). The rightward brown arrows indicate the putative promoters.

According to the gel electrophoresis results, the nucleic acid of AJO2 was degraded by DNase I, but undegraded by RNase A and mung bean nuclease, illustrating that the nucleic acid of AJO2 was double-strand DNA (dsDNA). Restriction endonuclease experiments showed that the genome had one *Spe*I restriction site. Therefore, genome digestion with *Spe*I would produce two fragments if it was linear and only one fragment if circular. The genome of AJO2 was confirmed to be linear by *Spe*I digestion with two bands produced (data not shown). The nucleotide sequence of AJO2 genome was determined with an average of 50× DNA sequencing coverage, and has been deposited in GenBank under accession numbers MH814913-MH814931. The genome of AJO2 consisted of 38,124 bp, with an average G+C content of 41 mol % (Figure 3). This was similar to the G+C content of host bacteria (41.4 mol %), suggesting that AJO2 was adapted to its host (Petrovski et al., 2011). Over 90% of AJO2 genome was represented by coding sequences. In total, 58 ORFs longer than 90 nucleotides were identified, including 18 ORFs on the positive strand and 40 ORFs on the opposite strand (Table 1). All ORFs were numbered consecutively. Thirty ORFs could be assigned predicted functions and 28 ORFs exhibited no significant identity to any hypothetical protein.

**Table 1:** Summary of ORFs and corresponding products in AJO2.

ORF^a^	Location^b^	Gene coordinate		Gene length (bp)	Protein function (conserved motif)^c^	*E*-value^d^
		Start	End			
1	–	2	100	99	Unknown	
2	–	102	971	870	Unknown	
3	–	1190	2305	1116	NTPases (cl17233)	1.00E-07
4	–	2308	2535	228	Putative DnaG-like primase (PHA02031)	9.00E-24
5	–	2537	3145	609	TOPRIM_primases (cl01029)	5.00E-06
6	–	3120	3470	351	Putative DnaG-like primase (PHA02031)	4.00E-07
7	–	3460	3723	264	Unknown	
8	–	3723	3866	144	Unknown	
9	–	4061	4204	144	Unknown	
10	–	4216	4731	516	Unknown	
11	–	4875	5414	540	Unknown	
12	–	5425	5680	256	Thioredoxin (cl00388)	3.00E-03
13	–	5984	6688	705	Unknown	
14	–	6777	7253	477	HTH_Tnp_4 super family (cl16321)	1.90E-03
15	–	7253	8341	1089	Unknown	
16	–	8397	8753	357	PHA 02046 super family (cl10354)	4.30E-11
17	–	8756	9088	333	Unknown	
18	–	9091	9675	585	T4-type Lysozyme (cl00222)	1.60E-15
19	–	9662	9988	327	BenE/Involved in benzoate metabolism (COG3135)	6.30E-04
20	–	10035	10460	426	Unknown	
21	–	10450	12030	1581	Collar/Phage tail collar domain (pfam07484)	7.30E-06
22	+	12029	12625	597	Unknown	
23	+	12618	13067	450	Recombination endonuclease VII (cl03794)	3.00E-10
24	+	13051	13242	192	Unknown	
25	+	13242	14177	936	Calcineurin-like phosphoesterase (pfam00149)	2.00E-05
26	+	14180	14824	645	Deoxynucleoside monophosphate kinase (cl14713)	2.20E-11
27	+	14829	16169	1341	DNA-directed RNA polymerase N-terminal (pfam14700)	5.00E-33
28	+	16290	17225	936	Unknown	
29	+	17228	17743	516	Calcineurin-like phosphoesterase (pfam00149)	4.00E-05
30	+	17876	18256	381	Unknown	
31	+	18259	19257	999	DNA-directed RNA polymerase N-terminal (cl20638)	5.00E-06
32	–	19403	20059	657	Unknown	5.30E-20
33	–	20144	20641	498	Unknown	
34	–	20649	20858	210	Unknown	
35	–	20875	21204	330	Hypothetical protein (cl10333)	
36	–	21176	21526	351	Hypothetical protein (cl10333)	4.20E-22
37	–	21943	24024	2082	DNA polymerase (cl02626)	7.40E-24
38	+	24378	25439	1062	Unknown	3.02E-21
39	+	25643	25939	297	Unknown	
40	+	25959	26258	300	Unknown	
41	+	26365	28189	1825	Phage_T7_tail super family (cl04321)	
42	–	28902	29045	144	Unknown	1.30E-06
43	–	29039	29587	548	DNA-dependent RNA polymerase (cl20211)	
44	–	30184	30381	198	Unknown	1.00E-38
45	–	30368	30487	120	Unknown	
46	–	30484	30654	171	Unknown	
47	–	30701	31900	1200	Head-tail connecting protein (cl19541)	
48	–	31926	32192	267	Tail fiber protein (cl04321)	8.00E-54
49	–	32195	32917	723	Putative internal core protein (cl20177)	6.00E-03
50	–	33462	33644	183	Hypothetical protein (PHA02030)	1.00E-16
51	+	33991	34921	930	Putative internal core protein (cl20177)	1.30E-04
52	+	34924	35682	758	Hypothetical protein (PHA02030)	1.70E-41
53	+	35913	36158	246	DUF1522 super family (cl06491)	1.70E-03
54	+	36270	36488	218	Putative membrane protein (cl01069)	3.00E-03
55	–	36489	37025	536	DNA breaking-rejoining enzymes (cl00213)	6.00E-24
56	–	37066	37329	263	Unknown	
57	–	37345	37605	261	Unknown	
58	–	37602	37973	372	uPA receptor	2.30E-03


*^*a*^ORFs were numbered consecutively.^*b*^+, positive strand; -, negative strand. ^*c*^Predicted function was based on amino acid identity, conserved motifs, and gene location within functional modules.^*d*^The probability of obtaining a match by chance as determined by BLAST analysis. Only values of less than 10^-*3*^ were considered significant.*

The genome of AJO2 was modularly organized into five categories, including DNA modification and regulation, RNA metabolism, cell lysis, structure morphogenesis and other potential functions. The first module (*orf* 21, *orf* 41, *orf* 47–49*, orf* 51, and *orf* 54) encoded proteins involved in phage head, tail and membrane morphogenesis. Interestingly, most of these genes were continuously distributed between 30,000 and 35,000 bp. A similar phenomenon has been reported that structure-related genes in tailed-phage genomes are generally clustered together and located behind the head protein genes (Brüssow and Desiere, 2001).

The region between *orf* 4 and *orf* 6 was predicted to participate in DNA replication. Two ORFs (*orf* 4 and *orf* 6) putatively encoded a DnaG-like primase (PHA02031), while *orf* 5 encoded a topoisomerase (PF01751). The Toprim (topoisomerase-primase) domain is a structurally conserved domain of ∼100 amino acids with two conserved motifs, one of which centers at a glutamate and the other at two aspartates (DxD). The glutamate is probably involved in catalysis, whereas the DxD motif is involved in the co-ordination of Mg (2+) required for the enzyme activity (Aravind et al., 1998). Deoxynucleoside monophosphate kinase (*orf* 27), DNA polymerase (*orf* 37), NTPases (*orf* 3) and recombination endonuclease (*orf* 23) were also revealed in the genome. The former three enzymes were related to DNA replication or repair, and the last one was related to DNA recombination. The *orf* 55 gene encoded a DNA breaking-rejoining enzyme, which was a phage integrase that mediated unidirectional site-specific recombination between two DNA recognition sequences, the phage attachment site (attP) and the bacterial attachment site (attB) (Groth and Calos, 2004). DNA-directed RNA polymerases were predicted to be encoded by *orf* 27 and *orf* 31. The N-terminal domain of DNA-directed RNA polymerase plays a role in the interactions with regions of upstream promoter DNA and nascent RNA chain, ensuring the processivity of the enzyme (Jeruzalmi and Steitz, 1998). The HTH_Tnp_4 super family putatively encoded by *orf* 14 might be a family of helix-turn-helix (HTH) motif, which has been found in all known DNA binding proteins that regulate gene expression. The HTH motif is highly similar in sequence and structure to repressor proteins, but it needs glycine to avoid steric interference (Brennan and Matthews, 1989). Such enzymes might be involved in the modification of the phage DNA.

Phage lysis modules typically consist of endolysin and holin genes that together are responsible for bacterial lysis and release of phage progeny (Daniel et al., 2007). In the AJO2 genome only the T4-type lysozyme was identified. The T4-type lysozyme processes a similar function to endolysins, which helps to release mature phage particles from the cell wall by hydrolyzing the 1,4-beta linkages between N-acetyl-D-glucosamine and N-acetylmuramic acid in peptidoglycan heteropolymers of prokaryotic cell walls. However, no ORF displaying identity to any known holin was detected within the AJO2 genome, suggesting that the presence of lysozyme may be sufficient for cell lysis, or that its functional mechanism is significantly different from previously identified lysin proteins. Mycobacteriophages have been reported to encode a novel mycolylarabinogalactan esterase (Lysin B), which facilitates lysis by compromising the integrity of the mycobacterial outer membrane linkage to the arabinogalactan–peptidoglycan layer (Payne et al., 2009).

Thioredoxin encoded by *orf* 12 is a small disulfide-containing redox protein that widely exists in all living organisms and helps to maintain a stable redox state (Holmgren, 1985). The putative gene *orf* 19 appeared to encode “BenE” protein, which possesses benzoate transmembrane transporter activity and was identified as a novel type of benzoate transporter (Saier, 2000). The last gene *orf* 58 encoded urokinase-type plasminogen activator (uPA) receptor, belonging to glycosyl-phosphatidylinositol (GPI)-linked cell-surface glycoproteins (Behrendt et al., 1991). In addition, some ORFs encoded functional proteins that were unusual in phage genomes. For example, calcineurin-like phosphoesterases (*orf* 25 and *orf* 29) contained motifs characteristic of a variety of enzymatically active phosphoesterases, but had previously only been identified in two human genes (Klabunde et al., 1996; Schwartz and Ota, 1997). The function of DFU1522 domain (*orf* 53) was unknown, and all of these proteins were from *Bradyrhizobium japonicum*.

Most putative genes were located close to each other with few intergenic spacer regions present. Regulatory sequences like promoters and terminators usually disperse in the space between genes (Lu et al., 2013). Eleven putative promoters were encoded in the AJO2 genome (Supplementary Table S3), and no terminators were identified using the FindTerm tool. No sequence homologs to excisionases or transposases were detected, further supporting the supposition that AJO2 is a virulent phage.

Eleven repeat structures were observed in the AJO2 genome, and these were designated R1–R11 (Table 2), including four long interspersed repeated sequences (LINES: R1, R2, R6, and R7), three long terminal repeats (LTR: R3–R5), three terminal repeats (TR: R8–R10) and a minisatellite DNA (also called variable number tandem repeat, VNTR). The repeat sequences occurred within genes or in intergenic regions. Two of these (R3 and R7) were located on one strand, and the rests were located on the opposite strand. All these sequences showed no similarity to any other and their functional role remains to be experimentally determined. In addition, no gene associated with pathogenicity was detected in the AJO2 genome, suggesting that AJO2 could be a potential candidate for phage therapy or control strategy against *A. johnsonii*.

**Table 2:** Repeats identified within the AJO2 genome.

Repeats	Type^a^	Scaffold	Coordinates		Content (%)	ORF(s)	Location^b^
			Start	End			
R1	LINE	2	10750	10810	0.1574	21	+
R2	LINE	2	11571	11622	0.1338	21	+
R3	LTR	2	11623	11670	0.1233	21	–
R4	LTR	2	11724	11806	0.2151	21	+
R5	LTR	2	11967	12008	0.1075	21	+
R6	LINE	4	16375	16438	0.1653	28	+
R7	LINE	8	26774	26826	0.1364	41	–
R8	TR	1	3616	4287	1.7600	7–10	+
R9	TR	4	18145	18297	0.3987	30–31	+
R10	TR	10	29691	29715	0.0630	44	+
R11	VNTR	2	11671	11723	0.1364	21	+


*^a^LINES, long interspersed repeated sequences; LTR, long terminal repeats; TR, terminal repeats; VNTR, variable number tandem repeat or minisatellite DNA.^*b*^+, positive strand; -, negative strand.*

### Phylogenetic and Similarity Analysis

**FIGURE 4:**
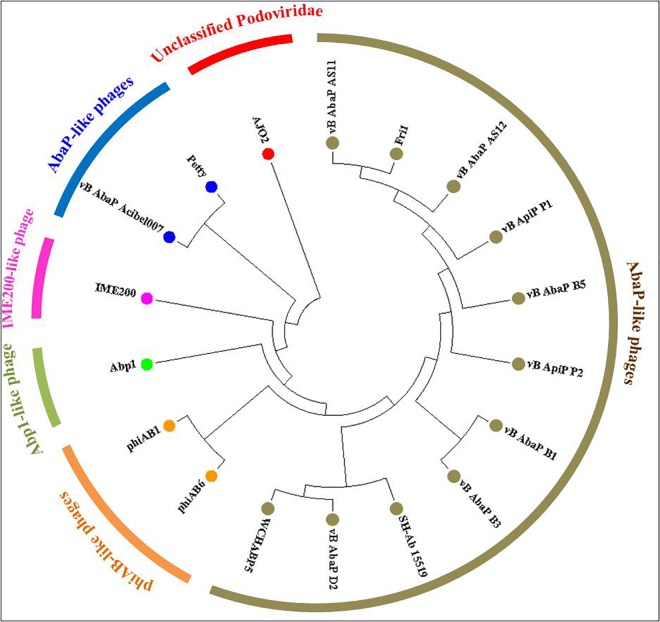
Phylogenetic relationships between phage AJO2 and 17 representative Acinetobacter phages with relatively close relationships according to BLASTn comparative results. The diagram was constructed by MEGA7 using the neighbor-joining method, and the different colors represent different phylogenetic branches, and phages with the same color show a close relationship.

**FIGURE 5:**
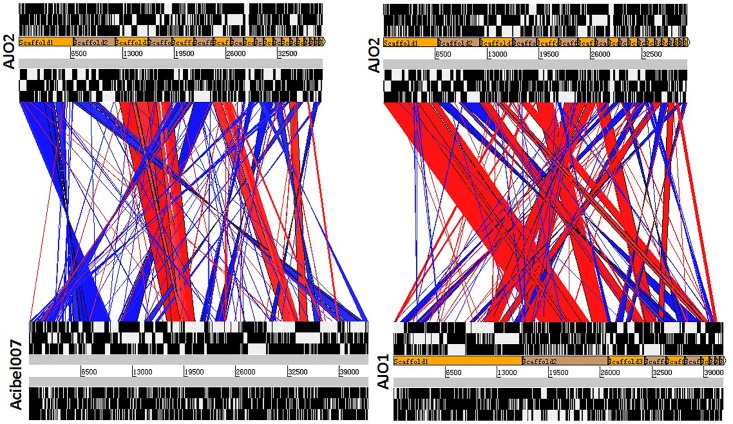
Comparative genome alignments of **(A)** phage AJO2 and Vb_AbaP_Acibel007, and **(B)** phage AJO2 and AJO1. Visual representation of the alignments (using tBLASTX) was carried out with the Artemis Comparison Tool (ACT, http://www.sanger.ac.uk/Software/ACT). The red bars spanning between the genomes represent individual tBLASTX matches, with inverted matches colored blue.

According to the genomic comparative analysis using the BLASTn algorithm, 17 Acinetobacter phages were detected with BLASTn scores above 100 (Supplementary Table S4). In particular, phage vB_AbaP_Acibel007 showed a high identity score of 91%, with a query coverage of 77%. As expected, AJO2 also had a relatively close relationship with vB_AbaP_Acibel007 in phylogenetic analysis (Figure 4). Among the 33 matches between vB_AbaP_Acibel007 and AJO2, the match with the highest score of 4774 bits located at 17,410–20,953 bp, which putatively encoded DNA-directed RNA polymerase. The highest identity similarity of 97% appeared between 23375 bp and 24592 bp, which region probably involved in encoding DNA polymerase. Screening 8 highly similar matches (score >1000, similarity >90%), most of them participated in DNA or RNA metabolism, and structure construction (Supplementary Table S5). The AJO2 genome sequence was also compared to that of vB_AbaP_Acibel007 using Artemis Comparison Tool (ACT) (Figure 5A). Although the extents of conserved regions between both phages were difficult to visualize by ACT, mainly because of multiple chromosomal rearrangements, their genomes still appeared quite different from each other.

It was notable that these phages with closer phylogenetic relationships shared a common host bacterium (*A. baumanii*), except for AJO2. The identification and adsorption between phage and host are mainly determined by phage tail structures (Lu et al., 2013). Generally, phylogenetically related phages have similar head structures, and cluster according to their capsid proteins (Bamford et al., 2005). However, three tail-related ORFs (*orf* 21, *orf* 41, and *orf* 48) in the AJO2 genome were unmatched or showed low similarity with other phages, suggesting that there could be differences among their host ranges. This was also in accordance with the previous host-range test result. In addition, the basic characteristics of phage vB_AbaP_Acibel007 and AJO2 were also different, such as host range and burst size (Table 3). Based on the above comparative analysis, both phages might descend from a common ancestor, but gradually diverged during evolution.

**Table 3:** Comparisons of phage AJO1, AJO2, and vB_AbaP_ Acibel007.

Features		AJO2	AJO1	Acibel007
Latent period (min)		∼30	30	21
Rise period (min)		50	40	30
Burst size		78.1	51.3	145
Classification		*Podoviridea*	*Podoviridea*	*Podoviridea*
Isolated host		*A. johnsonii* strain Pt405	*A. johnsonii* strain JN-11	*A. baumanii*
Genome size (bp)		38124	41437	42654
G+C %		41	41.15	41.2
Predicted genes		58	54	53
No. of genes with	Putative functions	27	22	23
	Unknown functions	31	32	30


sThe AJO2 genome displayed a higher similarity with that of the previously isolated phage AJO1 than vB_AbaP_Acibel007 (Figure 5B), thus differences in phage AJO2 and AJO1 were evaluated at multiple levels. By genomic comparison using the BLASTn algorithm, 13 matches were detected and distributed on 9 scaffolds with the total length of 17,772 bp (Supplementary Table S6). Although the identities were higher than 97%, query scores were quite low (≤11%). Most of these matched sequences putatively encoded structural proteins and RNA polymerases, which explained the similar morphology of both phages. As mentioned before, phages with similar tail structures might infect the same host. Further verifications of host range were performed and results showed that both phages were virulent to *A. johnsonii* strain Pt405 and *A. johnsonii* strain JN-11. However, their physiological features differed from each other, such as optimal MOI and burst size (Table 3). The genome size of AJO2 was smaller, but the content of genes with putative functions (46.6%) was higher than that of AJO1 (40.7%). Besides, plaques formed by the two phages were obviously different on DLA plates. AJO1 plaques were relatively smaller (2–4 mm) with clear edges, while AJO2 formed larger plaques (4–6 mm) with blurry edges (Supplementary Figure S3). Based on the above genomic and comparative analyses, AJO2 is a new member of the *Podoviridae* family. The comparative genomic analysis illustrated that bacteriophages are the most abundant and diverse biological entities, and it is hard to find homologous bacteriophages in the natural environment. Such successful evolutions of bacteriophages not only contribute to their diversity and survival, but also provide an advantage in infecting various host bacteria.

## Conclusion

This study describes the physiological characteristics and genome sequence of an *A. johnsonii* phage from bulking sludge. Bioinformatic analysis and comparison reveal that AJO2 is a novel lytic phage, with unique genomic features and relatively low similarity to other *Acinetobacter* phages. Moreover, this phage is highly specific to *A. johnsonii*. Therefore, it may be applied for the biocontrol of *A. johnsonii-*caused infection or other environmental problems.

It is known that bacteriophages play an important role in controlling their host bacteria, but the impact on the community structure in complex systems like activated sludge is still unclear. Since the genomic database of phages is extremely limited, isolation and sequencing of more phages from various environments are essential parts of further investigations. The newly isolated phage with genome sequence described here not only enriches the database of activated sludge phages, but also expands our understanding of various genomic features of phages. With the sequence data, it will be possible to develop specific primers and qPCR assays which could be used to follow their population dynamics in mixed systems. Based on the dynamic changes of both phages and host bacteria, their interaction mechanism and the function that AJO2 might have in sludge bulking may be uncovered. The evidence presented in this study contributes to a better understanding of the phages in activated sludge, but the complete genomes of more activated sludge phages clearly need to be analyzed before their ecological roles can be elucidated.

## Author Contributions

NF experimental design and operation and wrote the manuscript. MY revised the manuscript. RJ revised the manuscript. RQ helped to solve the problems during process operation, funding.

## Conflict of Interest Statement

The authors declare that the research was conducted in the absence of any commercial or financial relationships that could be construed as a potential conflict of interest.

## Footnotes

1 www.ncbi.nlm.nih.gov/projects/gorf

2 http://topaz.gatech.edu/

3 http://www.ncbi.nlm.nih.gov/Structure/cdd/cdd.shtml

4 http://pfam.sanger.ac.uk

5 http://linux1.softberry.com/berry.phtml

6 http://nc2.neb.com/NEBcutter2/

7 www.ncbi.nlm.nih.gov/projects/Sequin/
