# Supplementary material for: Isolation and Genomic Characterization of an Acinetobacter johnsonii Bacteriophage AJO2 From Bulking Activated Sludge
Source: Front Microbiol. 2019 Feb 20;10:266. doi: 10.3389/fmicb.2019.00266 (PMC6401600; doi:10.3389/fmicb.2019.00266)
Supplement: Supplementary file 1 [file Data_Sheet_1.PDF]

Frontiers in Microbiology

Isolation and genomic characterization of an *Acinetobacter johnsonii* bacteriophage AJO2 from bulking activated sludge

Niansi Fan[1, 2], Min Yang[2], Rencun Jin[1], Rong Qi[2*]

* Corresponding author. E-mail address: fns0219@aliyun.com. Postal address: Shuangqing Road No. 18, Haidian District, Beijing 100085, China.

1 College of Life and Environmental Sciences, Hangzhou Normal University, Hangzhou 310036, China

2 State Key Laboratory of Environmental Aquatic Chemistry, Research Center for Eco-Environmental Sciences, University of Chinese Academy of Sciences, Beijing 100085, China

Supplementary materials

Table A1 Bacterial strains used in this study.

Table A2 The host range of AJO2 using spot test method.

Table A3 Predicted promoters in AJO2 genome

Table A4 Comparison of phages with BlastN scores of more than 100 against the AJO2 genome.

Table A5 Highly similar matches between phage AJO2 and vB_ AbaP_Acibel007.

Table A6 Genome comparison between phage AJO2 and AJO1.

Fig. A1 One-step growth curve of phage AJO2.

Fig. A2 The host range of AJO2 using spectrophotometry.

Fig. A3 Plaques formed by (a) AJO2 and (b) AJO1 on double-layer plate.

Table A1 Bacterial strains used in this study.

Bacterial strain	Source	Culture condition	
		Medium[a]	Temperature (°C)
Acinetobacter johnsonii strain Pt405	Activated sludge	LB agar	30
Escherichia coli	Activated sludge	LB agar	30
Bacillus thuringiensis	Activated sludge	LB agar	30
Delftia tsuruhatensis strain M6	Activated sludge	LB agar	30
Bacillus methylotrophicus strain SY33	Activated sludge	LB agar	30
Flavobacterium	Activated sludge	LB agar	30
Kocuria rosea	Activated sludge	LB agar	30
Modestobacter versicolor strain CP153-2	Activated sludge	LB agar	30
Planococcus rifietoensis strain RL6	Activated sludge	LB agar	30
Acinetobacter sp. PHB1	Activated sludge	LB agar	30
Acinetobacter tandoii	CGMCC1.9078	Nutrient agar	30
Acinetobacter lwoffii	CGMCC1.9050	Nutrient agar	30
Acinetobacter junii	CGMCC1.8037	Nutrient agar	30
Acinetobacter haemolyticus	CGMCC1.4001	Nutrient agar	30
Acinetobacter calcoaceticus	CGMCC1.8824	Nutrient agar	30
Acinetobacter baumannii	CGMCC1.9049	Nutrient agar	30
Acinetobacter radioresistens	CGMCC1.15256	Nutrient agar	30
Acinetobacter bouvetii	CGMCC1.10395	Nutrient agar	30

[a] LB agar: 10 g L^{-1} tryptone, 5 g L^{-1} NaCl, 5 g L^{-1} yeast extract and 15 g L^{-1} agar; Nutrient agar: 5 g L^{-1} peptone, 3 g L^{-1} beef extract, 5 g L^{-1}NaCl and 15 g L^{-1} Agar; All media should be autoclaved at 121 °C for 15 min in advance.

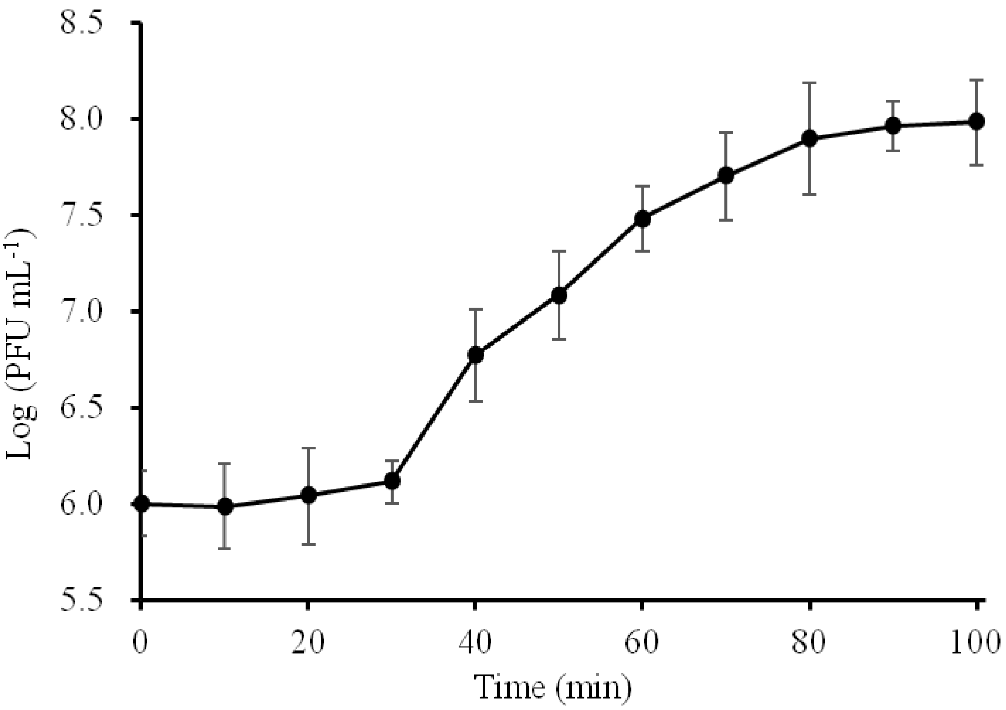

Fig. A1 One-step growth curve of phage AJO2.

Table A2 The host range of AJO2 using spot test method.

Tested bacteria	Phage Lysis[a]
Bacillus thuringiensis	N
Escherichia coli	N
Kocuria rosea	N
Modestobacter versicolor strain CP153-2	N
Flavobacterium	*N*
Bacillus methylotrophicus strain SY33	N
Delftia tsuruhatensis strain M6	N
Planococcus rifietoensis strain RL6	N
***Acinetobacter johnsonii* strain Pt405**	**Y**
Acinetobacter lwoffii	N
Acinetobacter junii	N
Acinetobacter haemolyticus	N
Acinetobacter calcoaceticus	N
Acinetobacter baumannii	N
Acinetobacter sp. PHB1	N
Acinetobacter tandoii	N
Acinetobacter haemolyticus	N
Acinetobacter radioresistens	N
Acinetobacter bouvetii	N

Y: plaques were observed; N: no plaques were observed.

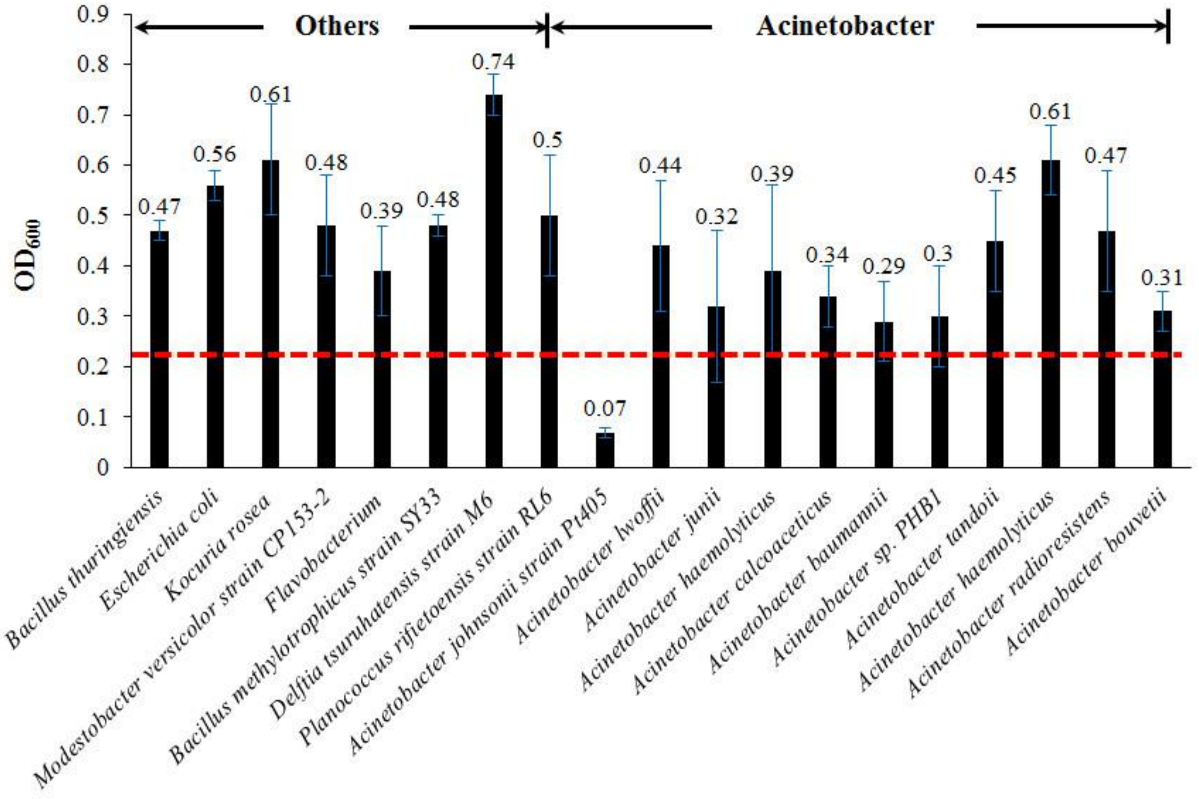

Fig. A2 The host range of AJO2 using spectrophotometry.

Table A3 Predicted promoters in AJO2 genome

Promoter	Start	End	Score[a]	Sequence
P1	1144	1189	0.99	ATGTGGTTTGAATACCTGTCTTACTGTTCTGTAATGCTGA**T**ACTGGTGGG
P2	5686	5731	0.98	ACTATCTTGAACAGTAGCACCACCTTTTATAATATTACCA**T**ACACGAGAA
P3	11365	11410	0.99	GGTTGATTGAATCTTCGCCCACATTGCAGGATAGTCAGCA**C**GATTAAGCA
P4	13821	13866	0.98	CCGTTTTTAACACCTGCTACAATCTGTGGTATCAACTTTG**T**ACACTTTGT
P5	15578	15623	0.99	AATTTTGGAAAACCCAAATGGCTGCTTGGTATACTAACGA**A**GCTAAACGT
P6	16159	16204	0.98	TTGATATTTAAAACTTACACAGTCCTCAGTACCATGCATG**A**TCATGGGCG
P7	18998	19043	0.99	AATTTTGGAAAACTCAAATGGCTGCTTGGTACACTAACGA**G**GCTAAACGT
P8	26307	26352	0.98	AAGATTTTAAATTTGAACGGGATATTTATTATAACTCGGA**T**GCGGATGCT
P9	33395	99440	0.99	CTATTGATTTAAGTTGTCAACCAATCTTTTAATATTTATT**C**ATTTAATTC
P10	33856	33901	0.98	TATTGATTTATATACTTATGCATATGTTATCTATTGTTAA**T**AAAGTACTG
P11	34957	35002	0.98	GAAGTTTTTAGACTTCTTACCAGTTACTTTACCATCGGCA**T**CTTTAACTT

a The score cutoff is 0.97. The transcription start is shown in larger and bolder font.

Table A4 Comparison of phages with BlastN scores of more than 100 against the AJO2 genome.

Phage	Source	Accession	Length	BlastN score	Query coverage	Identity	Reference
vB_AbaP_Acibel007	Belgium	KJ473423.1	42654	38301	77%	91%	Merabishvili et al., 2014
Petty	USA	KF669656.1	40739	604	2%	77%	Mumm et al., 2013
IME200	China	KT804908.2	41243	165	1%	72%	Unpublished
SH-Ab 15519	China	KY082667.1	40493	231	1%	72%	Unpublished
phiAB6	China	KT339321.1	40570	163	1%	72%	Lai et al., 2016
phiAB1	China	HQ186308.1	41526	231	1%	72%	Huang et al., 2013
vB_AbaP_D2	China	MH042230.1	39964	159	1%	71%	Unpublished
vB_ApiP_P2	China	MF033351.1	41514	154	1%	71%	Unpublished
WCHABP5	China	KY888680.2	40409	225	1%	72%	Unpublished
vB_AbaP_AS12	Russian	KY268295.1	41402	220	1%	72%	Unpublished
vB_ApiP_P1	Portugal	MF033350.1	41208	210	2%	71%	Unpublished
vB_AbaP_B3	Portugal	MF033348.1	40598	221	2%	71%	Unpublished
vB_AbaP_B1	Portugal	MF033347.1	40879	221	2%	71%	Unpublished
Abp1	China	JX658790.1	42185	216	2%	71%	Huang et al., 2013
vB_AbaP_B5	Portugal	MF033349.1	41608	216	2%	71%	Unpublished
vB_AbaP_AS11	Russian	KY268296.1	41642	205	2%	71%	Unpublished
Fri1	Russian	KR149290.1	41805	181	2%	70%	Unpublished

Table A5 Highly similar matches between phage AJO2 and vB_ AbaP_Acibel007.

Match	Score	Coordinates		Identity %	Strand	ORFs
		Start	End			
1	4774	18410	20953	91	+	*orf*31-35
2	4047	7352	10150	93	+	*orf*15-20
3	3367	11468	13235	95	+	*orf*21-24
4	2073	23375	24592	97	+	*orf*37-38
5	1507	19887	20889	94	+	*orf*32-35
6	1341	1793	2823	90	+	*orf*3-4
7	1336	6658	7473	96	+	*orf*14-15
8	1210	34085	35018	90	+	*orf*51-52

Table A6 Genome comparison between phage AJO2 and AJO1.

Match	Scaffold	Coordinates		Score	E value	Query %	Identity %
		Start	End				
1	11	30721	31938	2228	0	7	99
2	7	24378	25441	1914	0	11	99
3	7	25568	27381	1142	0	11	99
4	7	26202	28363	614	0	11	99
5	12	31938	32944	1842	0	6	99
6	14	33990	34922	1705	0	5	99
7	9	28902	30302	1269	0	6	100
8	9	28188	28421	425	0	6	100
9	9	29588	29658	130	0	6	100
10	18	37063	37622	1016	0	3	99
11	3	15918	20063	457	1.0E-129	1	100
12	4	19042	22096	300	2.0E-82	1	97
13	8	26539	26658	200	2.0E-58	0	100

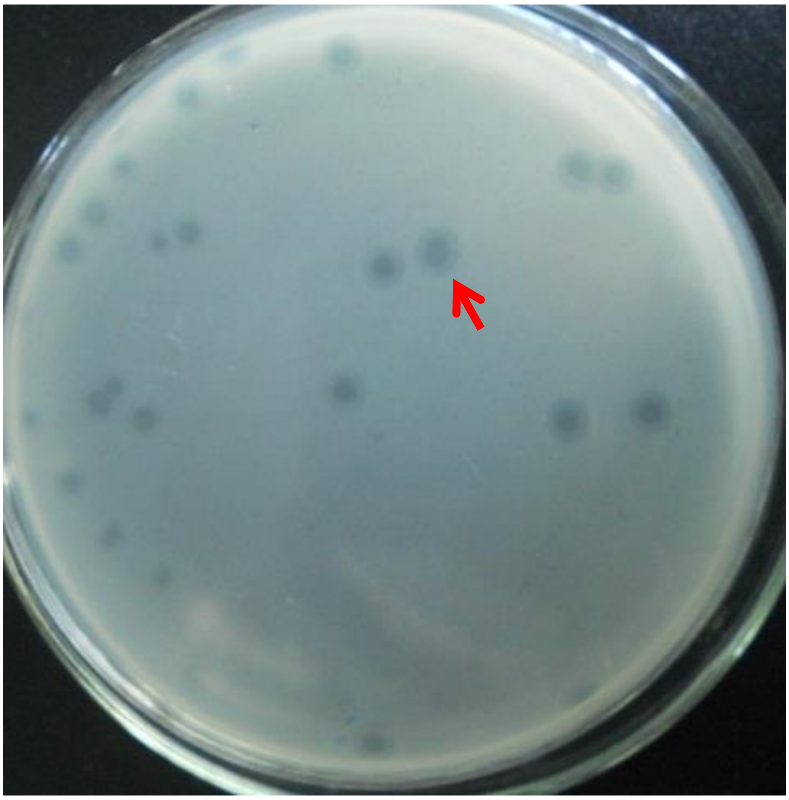

Fig. A3 Plaques formed by AJO2 on double-layer plate.
